# Universal health coverage in emerging economies: findings on health care utilization by older adults in China, Ghana, India, Mexico, the Russian Federation, and South Africa

**DOI:** 10.3402/gha.v7.25314

**Published:** 2014-10-31

**Authors:** Karl Peltzer, Jennifer Stewart Williams, Paul Kowal, Joel Negin, James Josh Snodgrass, Alfred Yawson, Nadia Minicuci, Liz Thiele, Nancy Phaswana-Mafuya, Richard Berko Biritwum, Nirmala Naidoo, Somnath Chatterji, on behalf of the SAGE Collaboration

**Affiliations:** 1 Human Sciences Research Council, Pretoria, South Africa; 2 Department of Psychology, University of the Free State, Bloemfontein, South Africa; 3 ASEAN Institute for Health Development, Mahidol University, Nakhon Pathom, Thailand; 4 Department Public Health and Clinical Medicine Epidemiology, Global Health Umeå University, Umeå, Sweden; 5 Research Centre for Gender, Health & Ageing, University of Newcastle, Newcastle, Australia; 6 World Health Organization, SAGE, Genève, Switzerland; 7 School of Public Health, University of Sydney, Sydney, Australia; 8 Department of Anthropology, University of Oregon, Eugene, OR, USA; 9 Department of Community Health, University of Ghana, Accra, Ghana; 10 National Research Council, Institute of Neuroscience, Padova, Italy; 11 Independent Consultant, Atlanta, GA, USA; 12 Office of the Deputy-Vice Chancellor, Nelson Mandela Metropolitan University, Port Elizabeth, South Africa; 13 World Health Organization, HIS/HSI, Genève, Switzerland

**Keywords:** health care use utilization, low- and middle-income, universal coverage

## Abstract

**Background and objective:**

The achievement of universal health coverage (UHC) in emerging economies is a high priority within the global community. This timely study uses standardized national population data collected from adults aged 50 and older in China, Ghana, India, Mexico, the Russian Federation, and South Africa. The objective is to describe health care utilization and measure association between inpatient and outpatient service use and patient characteristics in these six low- and middle-income countries.

**Design:**

Secondary analysis of data from the World Health Organization’s Study on global AGEing and adult health Wave 1 was undertaken. Country samples are compared by socio-demographic characteristics, type of health care, and reasons for use. Logistic regressions describe association between socio-demographic and health factors and inpatient and outpatient service use.

**Results:**

In the pooled multi-country sample of over 26,000 adults aged 50-plus, who reported getting health care the last time it was needed, almost 80% of men and women received inpatient or outpatient care, or both. Roughly 30% of men and women in the Russian Federation used inpatient services in the previous 3 years and 90% of men and women in India used outpatient services in the past year. In China, public hospitals were the most frequently used service type for 52% of men and 51% of women. Multivariable regression showed that, compared with men, women were less likely to use inpatient services and more likely to use outpatient services. Respondents with two or more chronic conditions were almost three times as likely to use inpatient services and twice as likely to use outpatient services compared with respondents with no reported chronic conditions.

**Conclusions:**

This study provides a basis for further investigation of country-specific responses to UHC.

A common and long-running concern across the world relates to the impact of increasing longevity on health care utilization (1, 2). This is a problem, particularly for low- and middle-income countries (LMICs) where 65% of the world’s population aged 60 and older currently resides, with this proportion expected to increase to 79% by 2050 (3). There are now strong ethical, political, economic and health arguments being discussed and debated in global forums regarding universal health coverage (UHC) (4). Complicating matters in LMICs is the fact that ill health is changing in character. Morbidity and mortality burdens from non-communicable diseases are exceeding burdens resulting from communicable diseases in an increasing number of developing countries (5).

Although these demographic and epidemiological transitions may not have as large an impact on overall health care use and costs as once predicted, there is an expectation that policy-makers will ensure that health and social systems are adequately equipped to support aging populations (6). The emergence of global interest in UHC may provide impetus to address these and other major policy challenges faced by governments in LMICs. For example, healthier aging cohorts and risk pooling under UHC may provide a path for sustainable health service financing and delivery (7). In the longer term, contemporary health care systems will need to provide at least a basic level of care for the increasing numbers of older citizens, ensure an equitable distribution of health care resources across all age groups, undertake structural and organizational changes to manage chronic conditions, and improve the co-ordination of care within existing systems (1).

Health care utilization among older adults in both LMICs and high-income countries is variously influenced by demographic, economic, and health status factors. Older women use primary care and community health services more than older men (8, 9), and older men use inpatient services more often than older women (10). Studies in a number of LMICs show higher inpatient utilization in urban compared with rural areas (9, 11). In China, researchers identified rural–urban disparity in health service use with rural respondents using physicians services less than urban respondents and hospitals more (12). Factors associated with health care utilization among older adults include older age (13), higher education, living alone, worse self-perceived health (14), chronic illness (15), and depressive symptoms (16), as well as the availability of accessible, affordable, quality health care (17, 18).

Evidence is necessary to inform the ongoing debate about longevity and healthy aging in both developed and developing countries. Although some data on health care utilization in older adults are available from LMICs, most of the research and policy attention has to date been in high-income countries. Sound evidence about health care utilization by older adults in LMICs is necessary to gauge the capacity of health care systems in LMICs to align with the UHC agenda.

The World Health Organization’s Study on global AGEing and adult health (SAGE) Wave 1 provides important standardized international data on health in older adults in six LMICs. The SAGE population represents 43% (700 million) of the global population aged 50 and older and 60% of those living in developing countries. The SAGE data allow comparative descriptions of national patterns of health care use in China, Ghana, India, Mexico, Russia, and South Africa. The objective of this study is to describe health care utilization and measure association between inpatient and outpatient service use and patient characteristics in these six LMICs.

## Methods

### Study population

SAGE is a longitudinal study with nationally representative samples of adults in China (2008/10), Ghana (2008/09), India (2007/08), Mexico (2009/10), the Russian Federation (2008/10), and South Africa (2007/08). All six SAGE countries used stratified multistage cluster sampling strategies with strata defined to ensure representation of a range of living conditions and urban and rural localities in each country. Face-to-face structured interviews based on standardized interviewer training procedures and survey instruments were used to collect household and individual level data. One household questionnaire was completed per household and all individuals aged 50-plus in selected households were invited to participate. Additional details about SAGE are provided elsewhere (19).

### Variables

The study population is described by: age groups (50–59, 60–69, 70–79, and 80-plus); sex (men and women); residence (urban vs. rural), and ordered quintiles of relative wealth. A random-effects probit model was used to estimate wealth levels based on asset ownership in each country (20). Estimates of asset-based wealth were applied to every household in each survey and used to establish quintiles of household wealth for each country. Quintile one represents the lowest (poorest) fifth of respondents in terms of their asset-based wealth and the wealthiest fifth of respondents is represented in quintile five. A variable indicating the number of possible chronic conditions was included in the regressions. This variable was categorized as - zero versus one versus two or more chronic conditions, these being arthritis, depression, asthma, and diabetes.

Self-assessed need for health care was ascertained by the question: ‘The last time you needed health care, did you get health care?’ Those who answered ‘yes’ were directed to further questions about their use of various types of inpatient and outpatient services, including long-term care, home care, or the use of traditional healers. Different time-frames were used in questions on inpatient and outpatient care in order to capture sufficient incident episodes and minimize recall bias (21). Inpatient use referred to a stay in a hospital and/or long-term care facility for at least one night in the previous 3 years. Outpatient use referred to ambulatory care received in the previous 12 months excluding an overnight hospital stay.

### Data analysis

Household-level and person-level analysis weights, based on the selection probability at each stage of sampling along with post stratification corrections, were applied to produce nationally representative cohorts (19). Age and sex standardizations based on WHO’s World Standard Population (22) were carried out to adjust for between-country age and sex differences.

This study presents weighted analyses of data self-reported by adults aged 50 and older from China, Ghana, India, Mexico, the Russian Federation, and South Africa. Individual country and pooled multi-country populations are described by age, sex, residence, and wealth quintiles for China, Ghana, India, Mexico, the Russian Federation, and South Africa. Only records with complete data on these socio-demographic variables were included. Subsequent analyses were undertaken on sub-samples (individual country and pooled) in order to describe responses to specific questions on health care use and need, types of services, and reasons for use. The denominators include only records with complete data for the questions of interest. Imputation methods were not used in this study.

Multivariable logistic regressions separately describe statistical association between socio-demographic factors, chronic conditions, country of residence, and inpatient and outpatient service use (as dependent binary outcome variables). The selection of covariates included in multivariable models was informed by the literature. P-values and variance inflation factors (VIF) are reported. All analyses were carried out using STATA version 11.1 (StataCorp, 2009).

### Ethics

SAGE has been approved by the World Health Organization’s Ethical Review Committee. In addition, each partner organization implementing SAGE obtained ethical clearance through their respective review bodies. Written informed consent was obtained from all study participants.

## Results

Table 1 gives summary statistics for the weighted study samples: China 13,096, Ghana 4,300, India 6,545, Mexico 2,304, the Russian Federation 3,937, South Africa 3,817, and pooled 33,798. There were 133 records excluded from the descriptive analysis because of missing data (61 from China, 5 from Ghana, 38 from India, 4 from Mexico, 5 from the Russian Federation, and 20 from South Africa). The percentage of men was low in the Russian Federation (39%) compared with China (50%), Ghana (53%), India (51%), and Mexico (47%). The proportion of adults aged 80 and over was 10% in Ghana compared with 5% in China and India, 9% in Mexico, 8% in the Russian Federation, and 6% in South Africa. Most respondents lived in urban areas in Mexico (79%), the Russian Federation (73%), and South Africa (65%). Mexico had the highest proportion of respondents in the wealthiest quintile (27%) and South Africa had the highest proportion in the poorest quintile (21%).

**Table 1:** Percent distribution of socio-demographic characteristics by country^a^ and pooled^b^, adults aged 50+, SAGE Wave 1

	China	Ghana	India	Mexico	Russian Fed	South Africa	Pooled
*N*	13,096	4,300	6,545	2,304	3,937	3,817	33,798
Age group	%	%	%	%	%	%	%
50–59	44.9	39.8	48.6	48.1	45.2	50.0	49.7
60–69	31.8	27.5	30.9	25.6	24.6	30.6	28.6
70–79	18.6	23.1	16.0	17.8	21.8	14.0	16.1
80+	4.6	9.7	4.5	8.6	8.4	5.5	5.7
Sex							
Men	49.8	52.5	51.0	46.8	38.9	44.0	48.8
Women	50.2	47.5	49.0	53.2	61.1	56.0	51.2
Residence							
Urban	47.5	41.0	29.0	78.8	72.7	65.0	44.2
Rural	52.5	59.0	71.0	21.2	27.3	35.1	55.8
Wealth quintiles							
Quintile 5 (highest wealth)	21.8	21.6	23.9	26.6	24.6	21.4	22.9
Quintile 4	23.4	20.7	19.6	16.6	20.5	19.8	21.9
Quintile 3	20.5	20.5	18.8	16.8	19.1	18.2	19.6
Quintile 2	18.1	19.1	19.5	24.7	19.6	19.9	18.8
Quintile 1 (lowest wealth)	16.3	18.2	18.2	15.3	16.2	20.7	16.9

a Individual country survey weights applied.

b Pooled country survey weights applied. Russian Fed=Russian Federation. Pooled=Pooled country data.

In the pooled multi-country analysis, 5% of men and 4% of women reported that they did not get health care the last time it was needed (Table 2). Across the countries, the proportions ranged from 1% in Mexico to 14% in Ghana.

**Table 2:** Percent of men and women who self-reported not getting health care the last time it was needed, country^a^ and pooled^b^, adults aged 50+, SAGE Wave 1

	China	Ghana	India	Mexico	Russian Fed	South Africa	Pooled
Total men *N*	4,951	2,001	2,886	854	1,072	1,272	12,881
Men (%)	5.9	14.2	2.1	1.1	8.9	1.5	4.9
Total women *N*	5,271	1,886	2,901	1,083	1,903	1,661	14,317
Women (%)	5.2	13.6	2.4	0.6	1.9	1.1	4.0

Denominators include only respondents who answered question on self-assessed health care.

a Individual country survey weights applied.

b Pooled country survey weights applied. Russian Fed=Russian Federation. Pooled=Pooled country data.

### Utilization of health care: type of facility

Respondents who reported getting health care the last time it was needed selected the types of services that they had used most frequently in the previous 3 years (Table 3). Relative to the other countries, private clinics and health care facilities were used frequently in India (by 38% of men and 42% of women), as were private hospitals (by 20% of men and 16% of women). Public clinics and health care facilities were used frequently in the Russian Federation (by 79% of men and also 79% of women) and public hospitals were used frequently in China (by 52% of men and 51% of women). The ‘other services’ category includes charity clinics and hospitals and traditional healers, and these services were frequently used in Ghana (by 27% of men and 25% of women).

**Table 3:** Types of health care services most frequently used in previous 3 years, by sex, country^a^, and pooled^b^, adults aged 50+, SAGE Wave 1

	China	Ghana	India	Mexico	Russian Fed	South Africa	Pooled
Men *N*	4,659	1,718	2,825	845	976	1,252	12,255
Women *N*	4,998	1,630	2,832	1,076	1,866	1,642	13,749
Private clinic, HC facility							
Men%	19.8	10.1	37.6	32.5	2.1	32.0	25.2
Women%	20.2	7.6	41.7	24.0	2.5	28.0	26.0
Private hospital							
Men%	1.0	7.0	19.7	3.8	0.1	4.1	7.0
Women%	1.4	6.3	16.2	8.6	0.2	1.6	5.8
Public clinic, HC facility							
Men%	22.0	19.2	5.0	51.3	79.0	45.0	20.8
Women%	22.9	24.9	6.1	54.6	78.5	53.4	24.8
Public hospital							
Men%	52.0	36.8	18.0	7.6	11.2	14.7	37.0
Women%	50.6	36.6	17.6	8.2	12.7	14.8	34.6
Other services not included							
Men%	5.3	27.0	19.7	4.8	7.6	4.3	10.0
Women%	5.0	24.7	18.5	4.6	6.1	2.2	8.7

Private clinics include private health care facilities excluding hospitals. Public clinics include private health care facilities excluding hospitals. Denominators include only respondents who self-reported getting HC the last time it was needed.

a Individual country survey weights applied.

b Pooled country survey weights applied. HC=Health care. Russian Fed=Russian Federation. Pooled=Pooled country data.

### Utilization of health care: inpatient and outpatient services

Table 4 shows inpatient and outpatient service use by those who reported getting health care the last time care was needed. In the pooled sample of over 26,000 older adults, almost 80% received inpatient or outpatient care, or both. Relative to the other countries, the use of inpatient services in the previous 3 years was high in the Russian Federation (28% for men and 30% for women). Past-year outpatient service use was high in India with 87% of men and 90% of women reporting use. In the multi-country sample, a similar proportion of men and women reported use of inpatient services in the previous 3 years (21% vs. 19%) but a higher proportion of women reported use of outpatient services in the past year (72% vs. 68%).

**Table 4:** Use of inpatient and outpatient services by sex, country^a^ and pooled^b^, adults aged 50+, SAGE Wave 1

	China	Ghana	India	Mexico	Russian Fed	South Africa	Pooled
Inpatient in the past 3 years^c^							
Men *N* (%)	4,676 (24.1)	1,733 (14.1)	2,827 (15.6)	845 (17.6)	1,118 (27.6)	1,308 (12.1)	12,380 (20.7)
Women *N* (%)	5,015 (21.7)	1,637 (11.0)	2,833 (13.7)	1,076 (10.7)	2,047 (29.2)	1,683 (11.4)	13,904 (19.3)
Outpatient in the past year^d^							
Men *N* (%)	4,666 (60.5)	1,723 (70.1)	2,828 (87.3)	845 (36.3)	1,119 (60.8)	1,314 (55.9)	12,366 (68.1)
Women *N* (%)	5,010 (64.6)	1,645 (77.1)	2,834 (90.4)	1,076 (44.3)	2,047 (69.8)	1,684 (67.2)	13,894 (72.3)
Inpatient, outpatient, or both^e^							
Men *N* (%)	4,696 (71.8)	1,740 (75.1)	2,829 (90.4)	845 (47.9)	1,119 (71.0)	1,335 (60.5)	12,418 (76.5)
Women *N* (%)	5,030 (73.8)	1,646 (80.6)	2,835 (92.9)	1,076 (49.5)	2,047 (79.0)	1,722 (68.9)	13,937 (79.1)

Denominators include only respondents who self-reported getting HC the last time it was needed.

a Individual country survey weights applied.

b Pooled country survey weights applied. Russian Fed=Russian Federation. Pooled=Pooled country data.

c Respondents who used inpatient services in the past 3 years.

d Respondents who used outpatient services in the past year.

e Respondents who used either inpatient services in the past 3 years, or outpatient services in past year, or both.

### Utilization of health care: reason(s) for use

Respondents who self-reported getting health care the last time it was needed (the denominator) self-selected main reasons for their use of health care services from a list of possible response options. Table 5 shows the top most common reasons for respondents’ last inpatient overnight hospital stay in the previous 3 years, and last outpatient service in the previous year. Common reasons for inpatient service use included problems with the heart (20% in the Russian Federation and 14% in China), generalized pain in Ghana (17%), acute conditions (17% in India), and communicable diseases in South Africa (13%). Common reasons given for outpatient service use were acute conditions (33% in China and 42% in India), generalized pain in Ghana (23%), and high blood pressure in Mexico (17%), the Russian Federation (25%), and South Africa (39%).

**Table 5:** Top most common reasons for last inpatient overnight hospital stay in previous 3 years, last outpatient service in previous year by country^a^, adults aged 50+, SAGE Wave 1

China	Ghana	India	Mexico	Russian Fed	South Africa
Inpatient stays					
Problems with heart (14.0%)	Generalized pain (16.9%)	Acute conditions (17.2%)	Surgery (17.6%)	Problems with heart (20.0%)	Communicable diseases (13.1%)
Surgery (10.1%)	Surgery (13.9%)	Surgery (13.2%)	Problems with heart (12.8%)	High blood pressure (17.4%)	Injury (13.1%)
High blood pressure (6.4%)	Communicable diseases (11.7%)	Problems with heart (5.6%)	Cancer (11.1%)	Surgery (14.0%)	High blood pressure (7.4%)
Injury (6.2%)	Acute conditions (11.0%)	Injury (6.6%)	Diabetes (10.5%)	Pain in joints (7.9%)	Stroke (7.4%)
Generalized pain (5.2%)	High blood pressure (6.5%)	Communicable diseases (5.5%)	Occupational (5.1%)	Problems with breathing (5.0%)	Generalized pain (7.0%)
Outpatient service					
Acute conditions (33.1%)	Generalized pain (23.0%)	Acute conditions (41.5%)	High blood pressure (17.2%)	High blood pressure (24.9%)	High blood pressure (38.8%)
High blood pressure (12.8%)	Communicable diseases (13.6%)	Pain in joints (10.2%)	Acute conditions (14.0%)	Pain in joints (12.7%)	Pain in joints (13.1%)
Pain in joints (9.2%)	Pain in joints (12.6%)	Generalized pain (9.6%)	Diabetes (13.7%)	Problems with heart (12.6%)	Diabetes (10.3%)
Generalized pain (5.3%)	Acute conditions (12.5%)	High blood pressure (6.7%)	Nutritional (9.9%)	Problems with mouth (11.2%)	Acute conditions (9.6%)
Problems with heart (4.9%)	High blood pressure (11.1%)	Problems with breathing (3.4%)	Pain in joints (5.7%)	Acute conditions (7.6%)	Problems with heart (3.5%)

Denominators include only respondents who self-reported getting health care the last time it was needed. Main reasons expressed as % of all responses to these questions within each country.

a Individual country age survey weights applied. Russian Fed=Russian Federation.

### Factors associated with inpatient and outpatient service use

In the crude model (not shown) compared with China, respondents in the Russian Federation were 40% more likely to use inpatient services (OR=1.4 95% CI: 1.1,1.7), respondents in Ghana were 50% less likely to use inpatient services (OR=0.5 95% CI: 0.4,0.6), and respondents in India (OR=0.6 95% CI: 0.5,0.7) and Mexico (OR=0.6 95% CI: 0.4,0.8) were 40% less likely to use inpatient services.

Table 6 shows the multivariable logistic regressions for inpatient and outpatient use. Inpatient service use was 50% higher in the 70–79 year age group (OR=1.5 95% CI: 1.3,1.7) compared with the 50–59 year age group. Women were significantly less likely to use inpatient services than men (OR=0.8 95% CI: 0.7,0.9). Respondents in the lowest wealth quintile were 20% less likely to use inpatient services compared with respondents in the highest wealth quintile (OR=0.8 95% CI: 0.7,1.0). Respondents with two or more chronic conditions were more than twice as likely to use inpatient services (OR=2.8 95% CI: 2.4,3.3) compared with respondents with no recorded chronic conditions. The VIF (1.04) was within acceptable range for collinearity.

**Table 6:** Logistic regression of factors associated with inpatient and outpatient service use, adults aged 50+, SAGE Wave 1

	Inpatient use *N=*26,177	Outpatient use *N=*154
	Odds ratio 95% CI	Odds ratio 95% CI
Country (Reference: China)		
Ghana	0.5 (0.4,0.6)***	1.6 (1.4,2.0)***
India	0.5 (0.4,0.6)***	4.4 (3.5,5.6)***
Mexico	0.5 (0.4,0.8)**	0.4 (0.3,0.6)***
Russian Federation	1.2 (0.4,0.8)	1.1 (0.8,1.5)
South Africa	0.4 (0.4,0.5)**	1.0 (0.8,1.5)
Sex (Reference: men)	1.0	1.0
Women	0.8 (0.7,0.9)***	1.2 (1.1,1.3)***
Age group (Reference: 50–59 years)	1.0	1.0
60–69	1.1 (1.0,1,3)	1.0 (0.9,1.2)
70–79	1.5 (1.3,1.7)***	1.2 (1.0,1.4)*
80+	1.2 (0.9,1.6)	1.1 (0.9,1.3)
Residence (Reference: urban)	1.0	1.0
Rural	1.1 (0.9,1.3)	1.4 (1.1,1.7)**
Wealth quintiles (Reference: highest wealth)	1.0	1.0
Quintile 4	1.0 (0.8,1.2)	1.0 (0.8,1.2)
Quintile 3	0.9 (0.8,1.1	0.9 (0.7,1.2)
Quintile 2	0.8 (0.7,1.0)	0.8 (0.6,1.0)*
Quintile 1 (lowest wealth)	0.8*	0.7 (0.5,0.8)**
Chronic conditions (Reference: none)	1.0	1.0
One chronic condition	1.8 (1.5,2.0)***	1.6 (1.4,1.7)***
Two or more chronic conditions	2.8 (2.4,3.3)***	1.9 (1.6,2.3)***

Denominator includes only respondents who received health care the last time it was needed. Inpatient use refers to the previous 3 years. Outpatient use refers to the previous 12 months. Pooled country survey weights applied.

*** *p*<0.01

** 0.01<*p*<0.05

* 0.05<*p*<0.1. Variance Inflation Factor for inpatient model=1.04. Variance inflation factor for outpatient model =1.11. CI=confidence interval.

In the crude model (not shown) with China as the reference country, respondents in India were almost five times more likely to use outpatient services (OR=4.8 95% CI: 3.9,6.0), respondents in Ghana were 60% more likely to use outpatient services (OR=1.6 95% CI: 1.4,2.0), and respondents in Mexico were 60% less likely to use outpatient services (OR=0.4 95% CI: 0.3,0.6).

Women were 20% more likely to use outpatient services than men (OR=1.2 95% CI: 1.1,1.3) and respondents in the 70–79 year age group were 20% more likely (OR=1.2 95% CI: 1.0,1.4) to use outpatient services than respondents in the 50–59 year age group (Table 6). Respondents in the lowest wealth quintile were 30% less likely to use outpatient services compared with respondents in the highest wealth quintile (OR=0.7 95% CI: 0.5,0.8). Respondents with two or more chronic conditions were almost twice as likely to use outpatient services (OR=1.9 95% CI: 1.6,2.3) compared with respondents with no recorded chronic conditions. The VIF (1.11) was within acceptable range for collinearity.

## Discussion

Overall, the use of health care services in the 3 years prior to SAGE interview varied by country. It is possible that differences in out-of-pocket payments influenced these findings to some extent. When out-of pocket-payments are high, people often delay or defer accessing or using services even if they believe they need care (23–25) . Out-of-pocket payments account for a large share of total health expenditure in LMICs (26). In 2010, out-of-pocket expenditure as a percentage of total health expenditure was 35% in China, 28% in Ghana, 62% in India, 47% in Mexico, 36% in the Russian Federation, and 7% in South Africa (27). Between 2007 and 2012, price adjusted out-of-pocket expenditure increased by 54% in China, 52% in the Russian Federation, 30% in Ghana, 24% in India, and 9% in South Africa. During the same period in Mexico, price adjusted out-of-pocket expenditure fell by 5% (27). The proportion of older adults in the SAGE countries who reported not getting health care the last time it was needed was higher in countries in which there was a higher proportion of out-of-pocket expenditure (Ghana and China) and relatively lower in countries with a smaller proportion of out-of-pocket expenditure (Mexico, South Africa, and the Russian Federation).

Although less than 5% of older adults in the SAGE countries self-reported not getting health care the last time it was needed, information on health care delivery systems, including out-of-pocket payments, doctor/patient ratios, types and location of facilities, is needed to further investigate this finding. The increasing prevalence of chronic diseases among older adults in LMICs (28–31) will impact on access to affordable health care as countries move toward UHC.

This study showed that the presence of one or more chronic health conditions was associated with higher outpatient and inpatient service utilization. Studies in Mexico, Ireland, Scotland, the United States, the Netherlands, Switzerland, South Korea, and Germany reported similar findings with multiple co-morbidity related to both higher utilization and costs for health care systems (11, 13, 32).

The main reasons self-reported for the use of inpatient and outpatient services in the SAGE countries is consistent with evidence from the WHO (33). The African countries continue to have a relatively high mortality burden due to communicable diseases, while in other LMICs the burden is relatively high for heart problems, high blood pressure, surgery and generalized pain.

Research undertaken in high-income countries shows that health care utilization peaks at about 80 years of age (34, 35). There was significantly higher inpatient and outpatient utilization in the 70–79 year age group in the SAGE countries.

Inpatient service use over the previous 3 years varied from 11% (for men) in South Africa to 29% (for women) in the Russian Federation. Outpatient service use over the previous year ranged from 36% (for men) in Mexico to 90% (for women) in India. A multi-country study of people aged 65 and older showed that the proportion using health care services varied from 6 to 82% among sites in Cuba, Mexico, Peru, Dominican Republic, Puerto Rico, Venezuela, China, India, and Nigeria (9). Two Brazilian studies, which investigated the use of primary care services by adults aged 60 and older in the previous 6 months, found that health care utilization ranged from 45 to 50% (15, 36). In a large nationally representative study conducted in China, 30% of adults aged 60 and older reported no physician visits in the past year (37).

Compared with older men, older women in the six SAGE countries used inpatient services less often and outpatient services more often. These results are generally consistent with evidence from studies conducted in high-income countries, and also with results from a multi-country study which included nine LMICs (8, 9, 13). In addition to gendered differences in service needs, social relations and norms also influence access to health care (38). Other studies have found that men are more likely to use emergency services and be admitted to hospitals compared with women (8, 10). It is also possible that men have fewer preventive health visits and delay accessing care when needed, which is consistent with men’s shorter life expectancies compared with women’s. It has also been suggested that, in terms of seeking health care, men and women react similarly to acute, life threatening, higher severity illnesses, but differently to chronic, less severe conditions (13).

Socioeconomic inequalities in health care use exist in most countries and in general, wealthier people are more likely to use health care services. The findings from this study support other evidence of positive association between wealth and use of health care in rich and poor countries (9, 38). Generally, economic factors, such as higher economic or wealth status, financial support, and health insurance, are associated with health care utilization among older adults (10, 13, 39). Older adults in lower income countries may not have access to financial support or pensions, making them more economically vulnerable (9, 40).

### Strengths and limitations

The results should be interpreted with caution. Self-reported ‘need’ may not represent true clinical need, many illnesses and chronic conditions are undiagnosed, and perceptions of health care need may be different in the SAGE countries.

Health care utilization depends on a range of factors that relate both to health care financing and delivery systems and to individuals themselves. We did not attempt to make adjustments for differences in health care delivery and financing in each of the SAGE countries. We acknowledge, however, that on the supply side, the structure and organization of health coverage and the location, mix, and types of public and private providers are all important factors, while on the receiving side, patients’ age, mental health, functioning, socioeconomic status, insurance status, proximity to care, perceptions of the quality and benefits of care, and levels of health literacy can influence decisions to seek care (41).

We were unable to report the extent to which cultural, contextual, economic, and structural factors, including health insurance, may have differently impacted the reporting of unmet need in this dataset. Most of the research in this area has been undertaken in high-income countries. Unmet need is an important indicator of health care access. These findings suggest a need for further research on unmet need in LMICs, particularly in light of the UHC agenda.

A major strength of this study is the use of standardized questionnaires individually administered by trained interviewers to large representative samples of adult populations in LMICs from different geographic regions of the world. Questionnaires were translated into local languages and all interviews were administered one-on-one in culturally appropriate settings. However, the cross-sectional nature of the Wave 1 data presents limitations with regard to inferring causality. When SAGE panel data become available for Wave 2 and beyond, it will be possible to investigate changes in health and health care utilization over time and also compare trends in use in different regions.

## Conclusions

Both rich and poor countries require practical guidance on ways to finance health systems and move toward UHC. No single policy mix will suit all countries. The findings add to current understanding of patterns of service utilization among adults aged 50 and older in six LMICs. This paper provides a basis for further research into health care access and need, both met and unmet, taking into account factors such as facilities, structures, systems of operation, and the mix of public/private investment.
